# An active alternative splicing isoform of human mitochondrial 8-oxoguanine DNA glycosylase (OGG1)

**DOI:** 10.1186/s41021-015-0021-9

**Published:** 2015-10-01

**Authors:** Chie Furihata

**Affiliations:** School of Science and Engineering, Aoyama Gakuin University, Sagamihara, Kanagawa 252-5258 Japan; Division of Molecular Target and Gene Therapy Products, National Institute of Health Sciences, Setagayaku, Tokyo 158-8501 Japan

**Keywords:** Human 8-oxoguanine DNA glycosylase, OGG1, Mitochondrial OGG1, OGG1-1a, OGG1-1b, OGG1-2a

## Abstract

Eight alternatively spliced isoforms of human 8-oxoguanine DNA glycosylase (*OGG1*) (*OGG1*-1a, −1b, −1c, −2a, −2b, −2c, −2d and −2e) are registered at the National Center for Biotechnology Information (NCBI). OGG1-1a is present in the nucleus, whereas the other seven isoforms are present in the mitochondria. Recombinant OGG1-1a has been purified and enzyme kinetics determined. OGG1(s) in mitochondria have not been fully characterized biochemically until recently. The major mitochondrial OGG1 isoform, OGG1-2a (also named β-OGG1), has also been expressed and purified; however, its activity is unresolved. Recently, we purified recombinant mitochondrial OGG1-1b and found that it was an active OGG1 enzyme. We reported its enzyme kinetics and compared the results with those of OGG1-1a. The reaction rate constant of OGG1-1b 8-oxoG glycosylase activity (*k*_g_) was 8-oxoG:C > > 8-oxoG:T > > 8-oxoG:G > 8-oxoG:A and was similar to that of OGG1-1a under single-turnover conditions ([*E*] > [*S*]). Both OGG1-1b and OGG1-1a showed high specificity towards 8-oxoG:C. The reaction rate constant of OGG1-1b *N*-glycosylase/DNA lyase activity (*k*_gl_) was 8-oxoG:C > 8-oxoG:T ≃ 8-oxoG:G > > 8-oxoG:A and that of OGG1-1a was 8-oxoG:C > 8-oxoG:T, 8-oxoG:G and 8-oxoG:A. The *k*_gl_ of OGG1-1b and OGG1-1a is one order of magnitude lower than the corresponding *k*_g_ value. OGG1-1b showed an especially low *k*_gl_ towards 8-oxoG:A. Comparable expression of *OGG1*-1a and *OGG1*-1b was detected by RT-PCR in normal human lung tissue and lung cell lines. These results suggest that OGG1-1b is associated with 8-oxoG cleavage in human lung mitochondria and that the mechanism of this repair is similar to that of nuclear OGG1-1a. Currently, the other five mitochondrial OGG1 isoforms have not been isolated. I summarize information on OGG1 isoform mRNAs, coding DNA sequences and amino acid sequences that are archived by the National Center for Biotechnology Information.

## Introduction

According to the National Center for Biotechnology Information (NCBI), the human 8-oxoguanine DNA glycosylase (OGG1) gene encodes the enzyme responsible for the excision of 8-oxoguanine (8-oxoG), a mutagenic base byproduct that occurs as a result of exposure to reactive oxygen (http://www.ncbi.nlm.nih.gov/gene/4968). 8-oxoG was first described in 1984 by Kasai et al. [1] and is an abundant DNA adduct caused by oxidative stress [2]. The action of OGG1 includes lyase activity for chain cleavage. In 1997 Aburatani et al. described four isoforms (OGG1-1a, −1b, −1c and −2) [3], and then in 1999, Nishioka et al. described seven isoforms (−1a, −1b, −2a, −2b, −2c, −2d and −2e) [4]. They classified these isoforms into two groups based on their last exon: type 1 isoforms end with exon 7 and type 2 isoforms end with exon 8. At present, their type 1 nomenclature cannot be applied to *OGG1*-1b because it contains only exons 1–6 (NCBI: NM_016819). Now, type 1 and type 2 *OGG1*s can be grouped with or without exon 8. Eight alternatively spliced isoforms of *OGG1* (*OGG1*-1a, −1b, −1c, −2a, −2b, −2c, −2d and −2e) are registered in the NCBI gene and nucleotide database. OGG1-1a is the only OGG1 present in the nucleus [4], whereas the other seven isoforms have been shown to be present in mitochondria [3–5]. Recombinant OGG1-1a has been purified and its enzyme kinetics determined [6–8]. Although mitochondrial OGG1 was suggested to have a crucial role against mitochondrial DNA damage [9], the responsible OGG1 splicing isoform(s) have not been described in detail. Recombinant production of the mitochondrial major OGG1 isoform, OGG1-2a (also named β-OGG1), has been carried out; however, the activity of this OGG1 was very low [9] or un-detectable [10]. Recently, we purified recombinant mitochondrial OGG1-1b and showed that it was an active OGG1 enzyme; we determined its enzyme kinetics, and compared these results with those of OGG1-1a [11]. Similar 8-oxoG glycosylase activity and *N*-glycosylase/DNA lyase activity was detected except for *k*_gl_ (*N*-glycosylase/DNA lyase activity) against 8-oxoG:A. Currently, the other five mitochondrial OGG1 isoforms have not been purified.

A review of all eight alternatively spliced isoforms has not been published; therefore, in this review I present the published data on mainly mitochondrial OGG1 isoforms and summarize the information on eight alternative splicing isoforms archived by the NCBI.

### OGG1-1b

Human *OGG1-*1b was cloned as an alternatively spliced isoform of *OGG1* by Abratani et al. in 1997 [3] and confirmed by Nishioka et al. [4]. They proposed that the *OGG1-*1b mRNA contained an extra 244 bp from intron 6 and the same exon 7 compared with the *OGG1-*1a mRNA. However, *OGG1-*1b mRNA is currently described in NCBI (NM_016819) as composed of 6 exons (exons 1–6) and does not possess exons 7 and 8. Localization of the OGG1-1b protein in mitochondria was published by Takao et al. [5]. They showed localization of a FLAG-tagged OGG1-1b in the mitochondria of COS-7 cells by immunofluorescence staining. The expression of *OGG1*-1b was demonstrated by RT-PCR in several human tissues, including lung [11, 12], colon [3], cerebrum [4], kidney [4], fetal brain [4], peripheral blood lymphocytes [13], and in human cell lines including normal-derived lung cell MRC-9, lung cancer cell lines, H23, H69, Lu65, Lu135, PC10 and PC13 [12], A549, ABC-1, EBC-1, LK-2, LU99 and RERF-LC-MA [11], Jurkat cells (a human T-cell leukemia cell line) [4], and an immortalized line of T-lymphocyte cells [14].

Recently, we purified recombinant OGG1-1b and OGG1-1a using commercial human lung total RNA as the starting material, and showed that OGG1-1b was an active OGG1 enzyme. We compared the enzyme kinetics of mitochondrial OGG1-1b with the nuclear OGG1-1a protein [11], as described in the next section.

### Comparison of enzyme kinetics between OGG1-1b and OGG1-1a

The reaction rate constants for 8-oxoG glycosylase activity (*k*_g_) and N-glycosylase/AP activity (*k*_gl_) were determined under single-turnover conditions ([E] > [S]) of OGG1-1a and OGG1-1b with 100 nM enzyme and 20 nM substrate [11]. Alexa 555-labeled 36-mer oligonucleotide substrates (8OG_36_Alexa: [Alexa 555]GGAATTCCTCGAGGT[8-oxoG]GACGGTATCCGATGGCCGCT, c-16C: AGCGGCCATCGGATACCGTC**C**ACCTCGAGGAATTCC, c-16A: AGCGGCCATCGGATACCGTC**A**ACCTCGAGGAATTCC, c-16G: AGCGGCCATCGGATACCGTC**G**ACCTCGAGGAATTCC and c-16 T: AGCGGCCATCGGATACCGTC**T**ACCTCGAGGAATTCC) were used. The *k*_g_ of the 8-oxoG glycosylase activity of both OGG1-1b and OGG1-1a was 8-oxoG:C > > 8-oxoG:T > > 8-oxoG:G > 8-oxoG:A (7.96, 0.805, 0.070, and 0.015 min^−1^, respectively for OGG1-1b, and 7.21, 1.37, 0.125, and 0.031 min^−1^, respectively for OGG1-1a). The enzymes show similar kinetic values. Both OGG1-1b and OGG1-1a showed high specificity towards 8-oxoG:C. The *k*_gl_ of OGG1-1b *N-*glycosylase/DNA lyase activity was 8-oxoG:C > 8-oxoG:T ≃ 8-oxoG:G > > 8-oxoG:A (0.286, 0.079, 0.040, and ~0.00 min^−1^, respectively) and that of OGG1-1a was 8-oxoG:C > 8-oxoG:T, 8-oxoG:G and 8-oxoG:A (0.254, 0.083, 0.075, and 0.072 min^−1^, respectively). The reaction rate constant of *k*_gl_ of OGG1-1b and OGG1-1a was one order of magnitude lower than that of their *k*_g_ values. OGG1-1b showed a particularly small *k*_gl_ towards 8-oxoG:A, and an exact numerical value of *k*_gl_ for OGG1-1b could not be calculated from the experimental conditions employed [11]. Similar multiple-turnover kinetics data (*A*_0_, *k*_obs_ and *k*_ss_) under [*S*] > [*E*] for OGG1-1b and OGG1-1a against the 8-oxoG:C substrate were observed. Similar substrate specificity of OGG1-1b and OGG1-1a against 8-oxoG:C and 8-oxoG:A was observed. Product formation was higher against 8-oxoG:C than 8-oxoG:A for OGG1-1b and OGG1-1a. APEX nuclease 1 (APEX1; NM_001641) was required to promote DNA strand breakage by OGG1-1b. These results suggest that OGG1-1b is associated with 8-oxoG cleavage in human lung mitochondria and that the mechanism of this repair is similar to that of nuclear OGG1-1a.

### Active site amino acids

Various amino acids in the active site of OGG1-1a have been proposed, including, Gly-42, Asn-149, An-150, Lys-249, Cys-253, Asp-268, Gln-315, Phe-319 [6], His-270, Gln-315, Asp-322 [15], Arg-154, Val-317, Phe-319 [10], Arg-46, Arg-131, and Arg-154 [16]. Hashiguchi et al. compared glycosylase activity of OGG1-1a and OGG1-2a by site-directed mutagenesis and suggested that Val-317 is a critical residue for glycosylase activity [10]. OGG1-1b protein is identical to OGG1-1a protein from amino acid 1 to 317, including Val-317, and is an active OGG1 [11] despite not possessing Phe-319 and Asp-322. OGG1-2a protein is identical to OGG1-1a protein from amino acid 1 to 316 but does not possess Val-317, Phe-319, or Asp-322 and its enzyme activity is low [9] or not detectable [10]. These results suggest that Val-317 is a critical residue for glycosylase activity. Other OGG1 isoforms have not been purified and their enzyme activities have not been determined.

### OGG1-2a

Human *OGG1*-2 (now *OGG1*-2a) was cloned as an alternatively spliced isoform of *OGG1* in 1997 by Abratani et al. [3] and Roldán-Arjona et al. [9]. Localization of OGG1-2a protein in mitochondria was demonstrated in COS-7 cells [4] and HeLa MR cells [3]. In addition, the expression of *OGG1*-2a was demonstrated by northern blot analysis and by RT-PCR in various tissues [3].

Inconsistent findings regarding OGG1-2a protein have been published. Hashiguchi et al. [10] purified recombinant OGG1-2a (β-OGG1) and reported that OGG1-2a did not show any significant OGG1 activity *in vitro*. They examined OGG1 activity with 100 nM OGG1-2a and 10 nM oligonucleotide as the substrate, and found no activity. In the control experiment, they examined 1 nM OGG1-1a and 10 nM oligonucleotide substrate and found active OGG1 activity. Roldán-Arjona et al. [9] reported the purification of recombinant OGG1-2a and showed OGG1 activity against 8-oxoG:C oligonucleotide with 1 μM enzyme and 5 nM substrate. The OGG1 activity of OGG1-2a in this experiment was very low, because they used an unusually high enzyme concentration.

Recently, Su et al. suggested that OGG1-2a (written as β-OGG1) was an accessory factor in mitochondrial Complex I function and was related to mitochondrial base excision repair [17].

### Other mitochondrial isoforms

*OGG1*-1c was cloned as an alternative splicing isoform of *OGG1* in 1997 by Abratani et al. [3]. Expression of *OGG1*-1c was demonstrated by RT-PCR in some human tissues including the colon [3]. Localization was demonstrated by expressing epitope-tagged OGG1-1c in COS-7 cells [5]. The expression of *OGG1*-2b, −2c, −2d, and −2e was demonstrated by RT-PCR in a small number of human tissues including the cerebrum and kidney, and in the Jurkat cell line by Nishioka et al. [4]. These proteins have not been purified.

### Analysis of information on the eight alternatively spliced isoforms of *OGG1* archived with the NCBI

Table 1 summarizes the mRNA accession number, nucleotide (nt) length, position of the 5′-UTR, coding DNA sequence (CDS) and 3′-UTR, exons, position of exon 1, 2, 3, 4, 5, 6, 7, and 8 of the eight alternative splicing isoforms of *OGG1*, as derived from the gene and nucleotide database of the NCBI (http://www.ncbi.nlm.nih.gov/gene/4968). Table 2 summarizes alternative splicing isoforms of *OGG1* CDS, nt length, identity to OGG1-1a CDS and identity to OGG1-2a CDS according to the NCBI and examined by BLAST (http://blast.ncbi.nlm.nih.gov/Blast.cgi). Table 3 summarizes the protein accession number, amino acid length, identity to the OGG1-1a protein, identity to the OGG1-2a protein (as determined by BLAST), position of mitochondrial and nuclear localization signals, and OGG1 activity.

**Table 1:** Alternative splicing isoforms of OGG1mRNA according to NCBI

Type	Name	mRNA	nt bp	5′-UTR	CDS	3′-UTR	Exons	Exon 1	Exon 2	Exon 3	Exon 4	Exon 5	Exon 6	Exon 7	Exon 8
		accession													
1	OGG1-1a	NM_002542	1652	1–343	344–1381	1382–1652	1,2,3,4,5,6,7	1–480	481–728	729–908	909–1090	1091–1241	1242–1291	**1292**–**1638**	No
1	OGG1-1b	NM_016819	1896	1–343	344–1318	1319–1896	1,2,3,4,5,6	1–480	481–728	729–908	909–1090	1091–1241	**1242**–**1882**	No	No
1	OGG1-1c	NM_016820	1669	1–343	344–1576	1577–1669	1,2,3,4,5,6,7	1–480	481–728	729–908	909–1090	1091–1241	1242–1291	**1292**–**1655**	No
2	OGG1-2a	NM_016821	2158	1–343	344–1618	1619–2158	1,2,3,4,5,6,8	1–480	481–728	729–908	909–1090	1091–1241	1242–1291	No	1292–2152
2	OGG1-2b	NM_016826	1957	1–343	344–1417	1418–1957	1,2,3,4,8	1–480	481–728	729–908	909–1090	No	No	No	1091–1951
2	OGG1-2c	NM_016827	1775	1–343	344–931	932–1775	1,2,3,8	1–480	481–728	729–908	No	No	No	No	909–1769
2	OGG1-2d	NM_016828	2258	1–343	344–1414	1415–2258	1,2,3,4,5,6,7,8	1–480	481–728	729–908	909–1090	1091–1241	1242–1291	**1292**–**1391**	1392–2252
2	OGG1-2e	NM_016829	2211	1–343	344–1312	1313–2211	1,2,3,4,5,6,7,8	1–480	481–728	729–908	909–1090	1091–1241	1242–1291	**1292**–**1344**	1345–2205

Bold columns show differences among isoforms

**Table 2:** Alternative splicing isoforms of OGG1 CDS to NCBI

Type	Name	mRNA									CDS		
		CDS in mRNA	From exon 1	From exon 2	From exon 3	From exon 4	From exon 5	From exon 6	From exon 7	From exon 8	nt length in CDS	identical to OGG1-1a CDS	Identical to OGG1-2a CDS
1	OGG1-1a	344-1381	344-480	481-728	729-908	909-1090	1091-1241	1242-1291	**1292-1381**	No	1038	—	1-949
1	OGG1-1b	344-1318	344-480	481-728	729-908	909-1090	1091-1241	**1242-1318**	No	No	975	1-951	1-949
1	OGG1-1c	344-1576	344-480	481-728	729-908	909-1090	1091-1241	1242-1291	**1292-1576**	No	1233	1-948, 966–1055 -1c: 949-1038-1a	1-948
2	OGG1-2a	344-1618	344-480	481-728	729-908	909-1090	1091-1241	1242-1291	No	**1292-1618**	1275	1-949	—
2	OGG1-2b	344-1417	344-480	481-728	729-908	909-1090	No	No	No	**1091-1417**	1074	1-748	1-748, 749–1074 -2c: 950–1275 -2a
2	OGG1-2c	344-931	344-480	481-728	729-908	No	No	No	No	**909-931**	588	1-569	1-569
2	OGG1-2d	344-1414	344-480	481-728	729-908	909-1090	1091-1241	1242-1291	**1292-1391**	**1392-1414**	1071	1-948	1-948
2	OGG1-2e	344-1312	344-480	481-728	729-908	909-1090	1091-1241	1242-1291	**1292-1312**	No	969	1-949	1-949

Bold columns show differences among isoforms

**Table 3:** Protein products of OGG1 isoforms according to NCBI

Type	Name	Protein accession	Amino acids	From exon 1	From exon 2	From exon 3	From exon 4	From exon 5	From exon 6	From exon 7	From exon 8	Identical to OGG1-1a	Identical to OGG1-2a	MTS	NLS	OGG1 activity
1	OGG1-1a	NP_002533	345	46^a^	83^a^	60^a^	60	51^a^	16	29	No	—	1-316	9 to 26	335-341	Active [6]
1	OGG1-1b	NP_058212	324	46^a^	83^a^	60^a^	60	51^a^	24	No	No	1-317	1-316	9 to 26	No	Active [11]
1	OGG1-1c	NP_058213	410	46^a^	83^a^	60^a^	60	51^a^	16	94	No	1-316	1-316	9 to 26	No	ND
2	OGG1-2a	NP_058214	424	46^a^	83^a^	60^a^	60	51^a^	16	No	108^a^	1-316	—	9 to 26	No	Unresolved [9, 10]
2	OGG1-2b	NP_058434	357	46^a^	83^a^	60^a^	60	No	No	No	108^a^	1-249	1-249, 250–357 -2b: 317–424 -2a	9 to 26	No	ND
2	OGG1-2c	NP_058436	195	46^a^	83^a^	60^a^	No	No	No	No	6^b^	1-190	1-190	9 to 26	No	ND
2	OGG1-2d	NP_058437	356	46^a^	83^a^	60^a^	60	51^a^	16	34^a^	6^b^	1-316	1-316	9 to 26	No	ND
2	OGG1-2e	NP_058438	322	46^a^	83^a^	60^a^	60	51^a^	16	6	No	1-316	1-316	9 to 26	No	ND

Bold columns show differences among isoforms

Abbreviations: *MTS* mitochondrial localization signal, RRMGHRTLASTPALWASI, *NLS* nuclear localization signal, KRRKGSK., *ND* not determined

^a^ indicates amino acids encoded cross a splice junction. ^b^ the same amino acid sequence. ^c^ the same amino aid sequence

The *OGG1*-1a mRNA has exons 1–7 and no exon 8. The CDS begins at nt 344 in exon 1, and the nt sequence of 1292–1381 (90 bp) in exon7 is the last part of the CDS. The *OGG1*-1a CDS is composed of part of exon 1, all of exons 2, 3, 4, 5, 6 and part of exon 7.

Exon 6 is the last exon of *OGG1*-1b mRNA. The nt sequence 1242–1318 (77 bp) in exon 6 is the last part of the CDS. Although the nt sequence of 1536–1882 (347 bp) in exon 6 of the *OGG1*-1b mRNA is the same as the entire exon 7 of the *OGG1*-1a mRNA (nt sequence 1292–1638, 347 bp), the former represents part of the 3′-UTR. As for the *OGG1*-1b CDS, the nt sequence of 1242–1291 from exon 6 of the *OGG1*-1b mRNA is identical to the whole exon 6 CDS of the *OGG1*-1a mRNA. The *OGG1*-1b mRNA nt sequence of 1292–1294 is identical to the first part of the exon 7 CDS of the *OGG1*-1a mRNA. The *OGG1*-1b mRNA 1295–1318 sequence (24 bp), which encodes seven amino acids and the stop codon, differs from the 1295–1318 sequence (CDS) from exon 7 of the *OGG1*-1a mRNA, resulting in a different amino acid sequence for the last seven amino acids of OGG1-1b when compared with the OGG1-1a sequence.

The *OGG1*-1c mRNA has exon 7, but the nt sequence of this exon is different from that of *OGG1*-1a mRNA. It also has no exon 8. The nt sequence 1292–1576 (285 bp) from exon 7 of the *OGG1*-1c mRNA is the last part of the CDS, but differs from the 1292–1381 CDS (90 bp) from exon 7 of the *OGG1*-1a mRNA. The nt sequence of 1309–1398 (90 bp) of the *OGG1*-1c mRNA, a part of the CDS from exon 7, is identical to 1292–1381 (90 bp) of the *OGG1*-1a mRNA, the entire exon 7 nt sequence.

Only type 2 *OGG1* mRNAs have exon 8. All type 2 *OGG1* mRNAs have the same exon 8 nt sequence (861 bp). The *OGG1*-2a mRNA has no exon 7. The nt sequence 1292–1618 (327 bp) in exon 8 of the *OGG1*-2a mRNA is the last part of the CDS and the sequence 1619–2158 (540 bp) is the 3′ UTR.

The *OGG1*-2b mRNA has no exons 5–7. The nt sequence 1091–1417 (327 bp) in exon 8 of the *OGG1*-2b mRNA is the last part of the CDS and is identical to the *OGG*1-2a CDS, resulting in an identical amino acid sequence for the last 108 amino acids of OGG1-2a and OGG1-2b.

The *OGG1*-2c mRNA has no exons 4–7. The nucleotide sequence 909–931 (23 bp: the first two nucleotides cross a splice junction from exon 3, plus six amino acids and the stop codon) from exon 8 of the *OGG1*-2c mRNA is the last part of the CDS, resulting in a different amino acid sequence from that of OGG1-2a and OGG1-2b. The nt sequence 932–1775 (844 bp) is the 3′-UTR and has a different length to the 3′-UTRs from the *OGG1*-2a (540 bp) and *OGG1*-2b mRNAs (540 bp).

The *OGG1*-2d mRNA has exons 7–8. The whole nt sequence of exon 7, 1292–1391 (100 bp), in the *OGG1*-2d mRNA is a CDS. The nucleotide sequence of 1392–1414 (23 bp: the first two nucleotides cross a splice junction from exon 7, plus six amino acids and the stop codon) from exon 8 of the *OGG1*-2d mRNA is the last part of the CDS, and gives rise to the same six amino acid sequence as that of the OGG1-2c protein. The nt sequence of 1415–2258 (844 bp) is the 3′-UTR.

The *OGG1*-2e mRNA has exons 7 and 8. The nucleotide sequence 1292–1312 (21 bp for six amino acids and the stop codon) from the first part of exon 7 of the *OGG1*-2e mRNA is the last part of the *OGG1*-2e CDS, resulting in an amino acid sequence that differs from the exon 7 CDS of *OGG1*-1a, *OGG1*-1c, and *OGG1*-2d. The nt sequence 1313–1344 is a part of the 3′-UTR. Exon 8 (861 bp) of the *OGG1*-2e mRNA is a continuous 3′-UTR. The whole exon 7 nt sequence 1292–1344 (53 bp) of the *OGG1*-2e mRNA is identical to part of the nt sequence 1339–1391 (53 bp) in exon 7 of the *OGG1*-2d mRNA.

## Conclusions

Eight alternatively spliced isoforms of human 8-oxoguanine DNA glycosylase (*OGG1*) are registered with the NCBI. OGG1-1a is present in the nucleus, whereas the other seven isoforms are present in mitochondria. Recombinant OGG1-1a has been purified and its enzyme kinetics studied. The mitochondrial major OGG1 isoform, OGG1-2a (also named β-OGG1), has been purified; however, the OGG1 activity of this enzyme was unusual and has not been determined. Recently, we purified recombinant mitochondrial OGG1-1b and showed that it is an active OGG1 enzyme. We reported its enzyme kinetics and compared the results with the corresponding kinetics of OGG1-1a. The OGG1 activity of OGG1-1b was similar to that of OGG1-1a, except for the *k*_gl_ against 8-oxoG:A. The *OGG1*-1b mRNA was detected by RT-PCR in normal human lung tissue and lung cells lines. These results suggest that OGG1-1b is associated with 8-oxoG cleavage at least in human lung mitochondria, and the repair mechanism is similar to that of nuclear OGG1-1a. Currently, the other five mitochondrial OGG1 isoforms have not been purified.

## Abbreviations

CDS: coding DNA sequence
5′-UTR: Five prime untranslated region
OGG1: Human 8-oxoguanine DNA glycosylase
nt: Nucleotide
NCBI: the National Center for Biotechnology Information
*k*_g_: the reaction rate constant of the 8-oxoG glycosylase activity
*k*_gl_: the reaction rate constant of *N*-glycosylase/DNA lyase activity
3′-UTR: Three prime untranslated region
